# Genetic, cellular, and environmental factors influencing tumourigenesis in carriers of succinate dehydrogenase germline mutations

**DOI:** 10.1530/ERC-26-0282

**Published:** 2026-09-04

**Authors:** Eugenie S Lim, Jean-Pierre Bayley, Anne-Paule Gimenez-Roqueplo, Roderick J Clifton-Bligh, Susan Richter

**Affiliations:** ^1^ Department of Endocrinology, St Bartholomew’s Hospital, Barts Health NHS Trust, London, United Kingdom; ^2^ Department of Endocrinology, William Harvey Research Institute, Queen Mary University of London, London, United Kingdom; ^3^ Department of Human Genetics, Leiden University Medical Center, Leiden, The Netherlands; ^4^ Université Paris Cité, Sorbonne Université, Inserm, Centre de Recherche des Cordeliers, Équipe Labellisée Ligue contre le Cancer, Paris, France; ^5^ Département de Médecine Génomique des Tumeurs et des Cancers, Fédération de Génétique et de Médecine Génomique, Assistance Publique-Hôpitaux de Paris Centre, Hôpital Européen Georges Pompidou, Paris, France; ^6^ Cancer Genetics Laboratory, Kolling Institute, University of Sydney, St Leonards, New South Wales, Australia; ^7^ Department of Endocrinology, Royal North Shore Hospital, St Leonards, New South Wales, Australia; ^8^ Auckland Cancer Society Research Centre, School of Medical Sciences, University of Auckland, Auckland, New Zealand; ^9^ Department of Cancer Sciences, School of Medical Sciences, University of Auckland, Auckland, New Zealand

**Keywords:** haploinsufficiency, tumour predisposition, genotype–phenotype, endogenous, exogenous

## Abstract

Succinate dehydrogenase (SDH) is an enzyme complex that plays a major role in cellular metabolism, as it sits at the interface between carbon metabolism in the Krebs cycle and oxidative phosphorylation for energy production. The precursor and product of the enzymatic reaction, succinate and fumarate, respectively, are regulators of various cellular and biological processes, such as epigenetic status, hypoxia responses and angiogenesis, metabolic reprogramming, tumourigenesis, and immune responses. Succinate is considered an oncometabolite, and *SDHx* genes are tumour suppressor genes. Carriers of germline pathogenic variants (PVs) in one of the *SDHx* genes (*SDHA*, *SDHB*, *SDHC*, *SDHD*, *SDHAF2*) carry a life-long risk of developing tumours, predominantly phaeochromocytomas and paragangliomas (PPGLs). Research has focused mainly on the disease state in which, in accordance with the Knudson two-hit model, the second allele is inactivated in tumour cells; the biological consequences are massive intracellular accumulation of succinate and/or reactive oxygen species (ROS), which in turn leads to tumourigenesis. Little is known about the haploinsufficient state, in which the wild-type *SDHx* copy at least partially sustains SDH function and keeps intracellular succinate and ROS levels in ranges that are, if not normal, then at least non-tumourigenic. This review will consolidate the literature regarding genotype differences between *SDHx* PV carriers, the phenotype of non-tumoural cells in healthy individuals, and the role of environmental factors in influencing tumour development, potentially via tipping succinate (or ROS) levels above a tumourigenic threshold.

## Part 1: inheritance patterns of *SDHx* PVs and disease penetrance

The SDH complex consists of four subunits encoded by the *SDHA*, *SDHB*, *SDHC*, and *SDHD* genes and utilises four assembly factors, only one of which, *SDHAF2*, is implicated in PPGLs (*SDHx*). Pathogenic variants (PVs) in one of the *SDHx* genes, causing loss of protein function, can give rise to tumour syndromes that show distinct differences in disease penetrance and clinical phenotype. Tumours that arise from SDH deficiency mainly include adrenal (phaeochromocytoma) and extra-adrenal paraganglioma of either sympathetic or parasympathetic nature (PPGL), and more rarely renal cell carcinoma (RCC), gastrointestinal stromal tumour (GIST), and pituitary adenoma/neuroendocrine tumour (PA/pitNET). Population prevalence of *SDHx* PVs is low, and incomplete penetrance leads to even lower prevalence of *SDH*-deficient tumours. PPGLs are catecholamine-producing tumours, and irrespective of an underlying genetic disorder, they are rare. Annual incidence of phaeochromocytoma is often quoted as 2–8 per million, but in practice, there has been no recent audit of this (1), and detection rates will vary greatly according to variations in healthcare practices and health economies globally. Detection rates have increased due to the development of next-generation sequencing methods, progress in identifying genetic causes, and implementation of cascade screening and surveillance.

Recognition of *SDHx* PVs as responsible for a tumour-predisposing syndrome is relatively new. Clinical genetic testing was developed at the beginning of the 21st century, together with national and/or regional expert centres for tumour screening and surveillance of *SDHx* PV carriers. Until recently, it has been assumed that inheritance followed a standard Mendelian pattern of 50% likelihood of inheriting a PV from a carrier parent. However, a recent study of multi-generational *SDHB* PV carrier families in Australia and the United Kingdom described a phenomenon of transmission ratio distortion from the expected 50% to 63% in favour of inheriting the PV from the parent (2). Transmission ratio distortion to 70% was also documented in a smaller group of 13 families with *SDHD* PVs, but *SDHA* or *SDHC* cohorts of sufficient size are not yet available for analysis.

Of all SDHx genes, penetrance for PPGLs is highest for a paternally inherited *SDHD* PV; while *SDHD* heterozygous PVs are transmitted by either sex of parent to a child of any sex, typically only paternally inherited *SDHD* PVs manifest disease, with a penetrance of up to 90% and an age of onset often in early adulthood (3). Head and neck (parasympathetic) paragangliomas (HNPGLs) are the most common, but sympathetic paragangliomas (i.e. located in the abdomen, pelvis, or mediastinum) are also observed. Frequent loss of the maternal copy of chromosome 11 in *SDHD*- and *SDHAF2*-related PPGLs (both genes are located on chromosome 11q) but not in PPGLs without a paternal inheritance phenotype (*SDHA*, *SDHB*, and *SDHC* are located on chromosomes 5p, 1p, and 1q, respectively), together with the presence of the major human imprinting centre on chromosome 11, suggests that the ‘Hensen model’ may apply in these cases (4). The Hensen model proposes that somatic loss of maternal chromosome 11 in individuals carrying a paternally derived PV in *SDHD* (or *SDHAF2*) eliminates not only the wild-type *SDHD* copy, but also an unknown maternally expressed, paternally imprinted modifier gene(s) in the chromosome 11p15 region, leading to SDH inactivation and tumourigenesis (5). It is very rare, evaluated at 5% or less, for a person with a maternally inherited *SDHD* PV to develop PPGL (6), which, according to the Hensen model, would require two additional independent somatic genomic events, namely recombination of the paternal 11q23 region with the maternal 11p15 region, together with subsequent loss of the recombined chromosome (6, 7).

*SDHB*-related PPGLs have a disease penetrance of approximately 30–40%, with sympathetic paraganglioma being the most common tumour type, but HNPGLs manifest frequently, and *SDHB*-deficient RCCs and GISTs are also recognised entities (8). Germline *SDHA* PVs show low penetrance, with fewer than 10% of all carriers developing PPGLs or GISTs (9). *SDHC*-related PPGLs have a lower overall penetrance than *SDHB*- and *SDHD*-related PPGLs and appear to have a predilection for HNPGLs. Of any *SDH*x-related tumour, *SDHB*-deficient PGLs have the highest likelihood of metastatic disease. This higher risk is likely due to secondary somatic events in tumour tissue, such as activation of telomerase and epithelial to mesenchymal transition (10); alterations in TERT or ATRX are strongly associated with metastatic *SDHB*-related PPGLs (11). Chromosomal gains and losses that are characteristic of SDH-related PPGLs are described above, but the relevant genes in these regions have not been identified. Incomplete penetrance of disease is a complex phenomenon and is likely attributable to the role of additional chromosomal regions that contain essential modifier genes; it may be influenced by possible genotype–phenotype correlations, with some PVs resulting in a high functional impact, while others have less effect on protein function. Classification of *SDHx* variants as pathogenic or non-pathogenic can be challenging and often requires specialised analyses of tumour tissue, which may be unavailable, often leading to uncertainty in the case of variants that lack sufficient evidence for a definitive pathogenic or likely pathogenic (P/LP) classification (12). Data collected from cohorts of families of PVs are better able to capture penetrance compared with PVs that are less well represented in large family groups, perhaps identified at the time of a tumour and without cascade screening being available (or the family not available for screening). Current recommendations propose lifelong surveillance for all subjects carrying an *SDHx* P/LP variant, but relatively low penetrance (excepting *SDHD*) means a majority of carriers who will never develop SDH-related disease will still be subjected to intensive surveillance. There is a growing need to balance benefits of early detection against cost-effectiveness and psychological impacts, potentially using more personalised risk-based surveillance.

**Figure 1:**
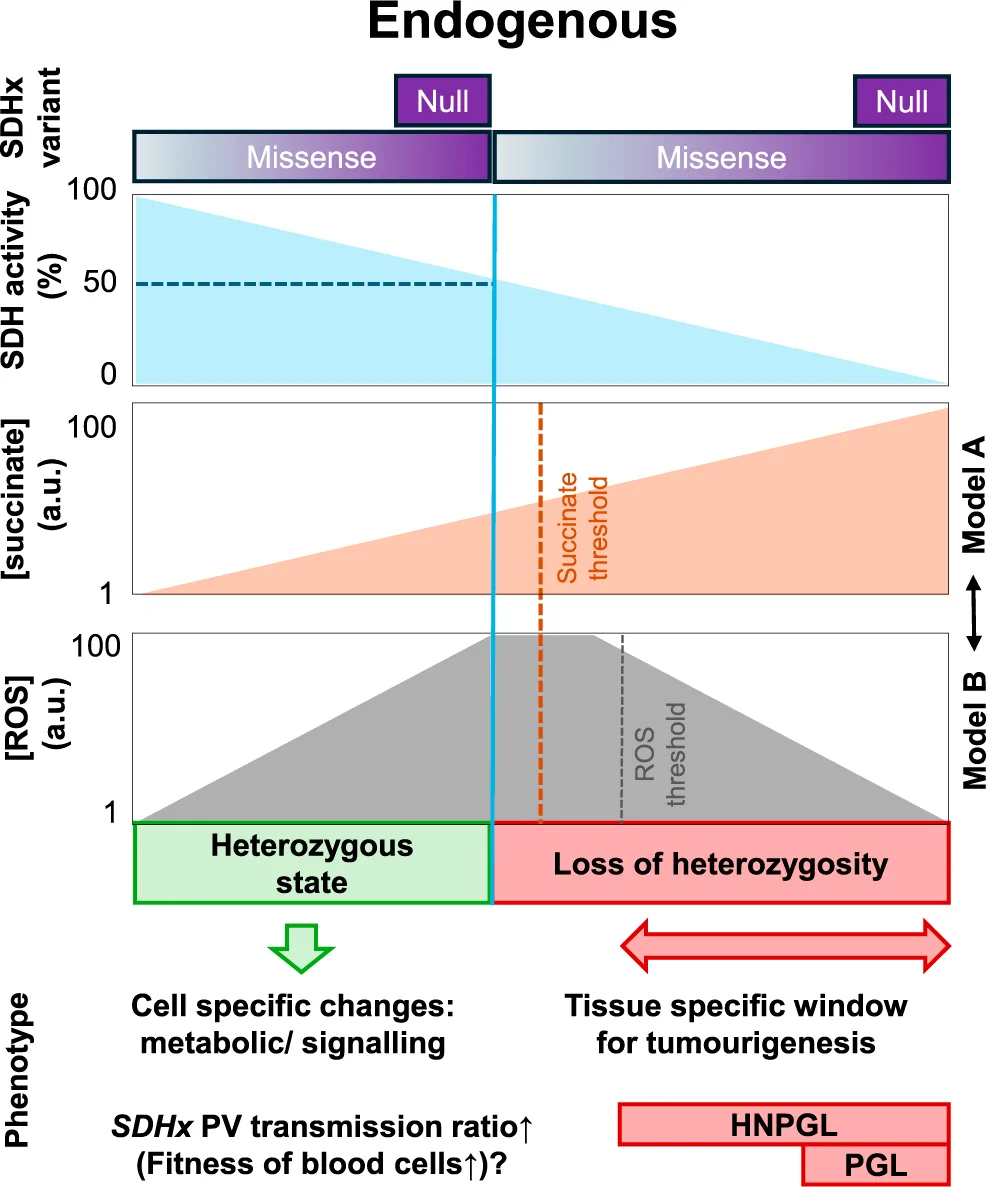
Model depicting endogenous factors influencing tumourigenesis in *SDHx* PV carriers. Left: in the heterozygous state, partial disruption of SDH activity causes cell-specific alterations. Right: models A and B are shown according to (14) and describe tissue-specific susceptibility to tumourigenesis. A combination of both may be conceivable with high succinate (model A) being the prerequisite and ROS sensitivity being the constraint (model B). Model A: SDH loss leads to the accumulation of high levels of succinate and inhibition of α-ketoglutarate-dependent enzymes, the likely driver of tumourigenesis in PPGLs. Truncating (null) variants cause significantly higher succinate levels than missense variants; levels high enough to cause tumour initiation in both sympathetic (PGL) and parasympathetic (HNPGL) tissues. Conversely, as missense variants may not abolish succinate oxidation completely, succinate levels in most cell types are too low to cause tumour initiation. For partially active missense variants, the ‘succinate tumourigenic threshold’ may only be reached in highly metabolically active cells, such as parasympathetic paraganglia. Model B: if certain *SDH* missense variants maintain residual activity of the SDH enzyme complex, then with ongoing succinate oxidation, this will lead to very high ROS levels due to disruption of the electron flow pathway to the normal electron acceptor, ubiquinone. High ROS levels may in turn lead to ROS-mediated toxicity in vulnerable sympathetic PGL precursor tissues, and perhaps in a broader range of tissues, explaining the tissue specificity of SDH variants. Sympathetic paraganglia and other tissues likely have weaker anti-oxidative defences compared with oxygen-sensing parasympathetic tissues, which likely require tight redox control due to the direct involvement of ROS in oxygen-sensing, explaining the association of missense variants with HNPGLs. In this scenario, low-ROS truncating (null) variants cause tumours in sympathetic tissues (PGL), whereas ROS-mediated toxicity driven by missense variants limits tumourigenesis in these tissues. ROS therefore functions as an inhibitor of tumourigenesis in sympathetic tissues, with missense SDH gene variants acting as anti-oncogenic tumour suppressors.

It is well documented that *SDH*-related PPGLs differ in primary clinical risk of thoracic–abdominal–pelvic PPGLs (*SDHB*) and HNPGLs (*SDHD*, *SDHAF2*, and *SDHC*), but recent studies are beginning to reveal clinically significant correlations between certain types of variants as well as specific variants, and clinical presentation (13, 14). For instance, *SDH*x variant types, i.e. missense variants vs protein-truncating variants (PTVs), seem to have distinct effects on PPGL development, but a largely comparable effect on HNPGLs, suggesting biological differences between precursor tissues. Furthermore, the Dutch founder variant *SDHD* p.(Asp92Tyr) confers a significantly higher risk of PPGLs compared with *SDHD* variant p.(Pro81Leu) or p.(Tyr114Cys), suggesting that allelic-specific differences may be relevant in certain contexts. Hypothetical models have been proposed to explain these variant-specific effects on disease development (15). They suggest that *SDHx* variants cause or prevent tumour initiation in specific precursor cell types because corresponding tissues have higher or lower tolerances to metabolic disruptions caused by these *SDHx* variants (Fig. 1). Furthermore, *SDHx* P/LP variants differ in their impact on SDH activity, ranging from complete loss of function (e.g. from protein-truncating variants) to retaining a small amount of residual function (e.g. for some missense P/LP variants); truly hypomorphic alleles resulting in only modest functional impact may not be severe enough to cause PPGLs, but can be associated with the autosomal recessive disorder Leigh syndrome (20).

Dysfunctional SDH is a major source of reactive oxygen species (ROS), but ROS production is inhibited when intracellular succinate levels increase (16). One model proposes that *SDHx* missense variants disrupt electron flow, leading to ROS levels that are toxic in sympathetic PPGL precursor cells but well controlled in oxygen-sensing parasympathetic paraganglion cells (HNPGLs). Alternatively, a succinate threshold may limit or allow tumour initiation. In this model, *SDHx* variants that completely abolish SDH activity cause succinate accumulation to a high enough threshold to initiate PPGL tumourigenesis *per se* via disruption of α-ketoglutarate-dependent enzymes. By contrast, missense variants that result in less succinate accumulation may cause tumours only in the metabolically active parasympathetic paraganglia that naturally have higher levels of succinate. Preliminary evidence supporting this model has been published recently (17).

## Part 2: phenotypic adaptations in haploinsufficient *SDHx* cells

*In vitro* studies have focused largely on cancer cells, with complete or partial loss of SDH function resulting in succinate elevation akin to that present in tumours from patients with *SDHx* pathogenic variants (PVs). This accumulation of succinate acts as an oncometabolite in driving tumour progression and invasiveness, predominantly mediated by inhibition of α-ketoglutarate-dependent enzymes, such as prolyl hydroxylases that regulate hypoxia-inducible factor (HIF) stability, and of DNA and histone demethylases, resulting in pseudohypoxic signalling and epigenetic reprogramming, respectively (18, 19). Altered metabolic function via enhanced glycolysis, dependencies on pyruvate and glutamine anabolism, and changes in iron metabolism were also reported (20, 21). Partial loss of SDH complex activity in cancer cell lines has shown similar features, although smaller increases in succinate were present compared with that seen in cells with complete loss of SDH activity (22, 23, 24, 25).

Few studies focus on SDH loss in non-cancerous tissues. Like *SDHx* PV carriers who do not present with clinical symptoms, zebrafish with partial loss of *sdhb* were equally viable, with even a trend towards increased lifespan, and small increases in succinate levels were measurable (26). This indicates that the wild-type gene copy in carriers is not sufficient to maintain normal SDH function, but the extent to which small increases in succinate contribute to tumourigenesis is unclear. Currently available data in conditional *Sdhb* knockout mice suggest that partial (single-allele) loss of *Sdhb *is insufficient to drive tumour formation, indicating that additional drivers for proliferation are required (27). Cell models largely support this notion, as SDH deficiency causes delayed rather than accelerated growth, although differences between cell types exist (23, 24). In humans, such additional drivers are suspected to relate to cellular hypoxia, pseudohypoxia, altitude (28), and potentially other environmental factors, such as pesticides that inhibit SDH (29).

Elevated circulating succinate in *SDHx* carriers without a tumour depends upon which SDH subunit is mutated. Carriers of *SDHB* PVs have generally higher plasma or serum succinate levels than carriers of *SDHC* or *SDHD* PVs (30, 31). Whether higher circulating succinate levels contribute to increased tumour risk is unclear; however, the lack of complete penetrance for carriers of *SDHB* PVs argues against this hypothesis. On the other hand, more severe inhibition of the succinate complex caused by PVs in *SDHB* compared with *SDHC* or *SDHD* appears to be connected to more aggressive progression once a tumour has formed.

As more detailed observations in specific cell types become available, a more granular view of biological adaptation to *SDHx* haploinsufficiency emerges. Metabolic analyses of the three main blood cell types (neutrophils, mononuclear cells, and erythrocytes) revealed very different metabolic adaptations to loss of one *SDHx* allele (Table 1). Neutrophils of *SDHB* PV carriers show higher levels of succinate and higher levels of lactate, indicating a shift towards higher glycolytic rates, compared with normal controls (32). Conversely, peripheral mononuclear cells (PBMCs) of carriers with *SDHx* PVs do not have elevated succinate, succinate:fumarate, or lactate levels (30). As high intracellular succinate levels induce a pro-inflammatory phenotype in macrophages (M1), it is conceivable that succinate levels are more tightly controlled in these cells (33). The M1 state is accompanied by a metabolic switch from oxidative phosphorylation to glycolysis. At the same time, extracellular succinate acts as a messenger molecule via the receptor SUCNR1, promoting anti-inflammatory responses (M2) (34). This highlights the multifaceted roles that succinate plays in different settings, and it suggests that high numbers of M2 macrophages in tumours of *SDHx* carriers may be caused by excessively high levels of succinate produced by tumour cells (35).

**Table 1:** Metabolic adaptions in blood cells of *SDHx* PV carriers.

	Metabolic changes in *SDHx* PV carriers compared with non-carriers			References
	Erythrocytes	PBMCs	Neutrophils	
Succinate	-	-	↑	(30, 32)
Lactate	↑	-	↑	
Physiological implications	May contribute to enhanced fitness, analogous to hypoxia response to high altitude	Succinate signalling drives inflammatory responses in T cells and monocytes; hence, levels may be tightly regulated	Enhanced survival	

Additionally, SDH function is essential for the fitness of CD4^+^ and CD8^+^ T cells and the production of inflammatory cytokines (36), supporting the suggestion of more stringent metabolic control in PBMCs. Upon activation, T cells upregulate glycolysis and Krebs cycle function. When SDH is inhibited, T cell activation is less efficient due to decreased respiratory function. Taken together, *SDHx* haploinsufficiency appears largely compensated without pathological effects on either innate or adaptive immunity. However, specific data for carriers, e.g. on frequency and length of infections, or cellular studies testing T cell activation, are not available.

Unlike other cell types, mature erythrocytes do not have mitochondria, but SDH subunits are present in the membrane fraction (37). Although erythrocyte succinate levels are comparable between *SDHx *carriers and carriers of PVs in other PPGL susceptibility genes, the lactate:α-ketoglutarate ratio, as a marker for heightened glycolysis, was elevated in SDHx carriers (30). A similar phenotype of hyperglycolysis was described in erythrocytes from people who spend time at high altitudes (38), consistent with metabolic adaptation to hypoxia. This analogy suggests that succinate-mediated stabilisation of HIFs is responsible for increased glycolysis in erythrocytes from carriers of *SDHx* PVs. To date, it is unclear whether this hyperglycolytic state has adverse or even beneficial effects for the health of people carrying *SDHx* PVs. As succinyl-CoA is one of the precursors for heme biosynthesis, higher succinate levels in the liver may support a metabolic adaptation to hypoxia.

Data on *SDHx* haploinsufficiency in cell types other than haematopoietic cells are not available. Recent studies, however, point towards a major role of succinate during embryonic development and pregnancy maintenance (reviewed in (39)). Epigenetic regulation by succinate was shown to drive transitions between cell types during early embryonic development (18). On the one hand, succinate can promote a more stem-like naive state (40) that is also observed in tumours with SDH deficiency. On the other hand, succinate can drive cells towards a neuroectodermal lineage (41). Not only epigenetic signalling but also activation of SUCNR1 by extracellular succinate has been shown to regulate pluripotency (40). In addition, succinate production by trophoblasts is essential for establishing pregnancy, as low levels are linked to spontaneous abortions (42). Potential elevated succinate production in trophoblasts with *SDHx* PVs may explain the observed distortion of transmission of mutated *SDHB* and *SDHD* alleles (2). Furthermore, succinate in the umbilical cord promotes placental angiogenesis through SUCNR1 signalling (43). This process becomes important to counteract hypoxic episodes, e.g. during labour.

These fundamental roles of succinate, in combination with limited data on haematological cells in *SDHx* PV carriers, suggest that even modest changes in production levels caused by haploinsufficiency might have biological consequences (Fig. 1). The magnitude and direction of these effects, however, remains unclear and may be either adaptive (beneficial) or maladaptive (harmful). Whether any of the described metabolic changes in blood cells of PV carriers play a role in tumour development will be part of future investigations.

## Part 3: environmental influences on disease initiation

**Figure 2:**
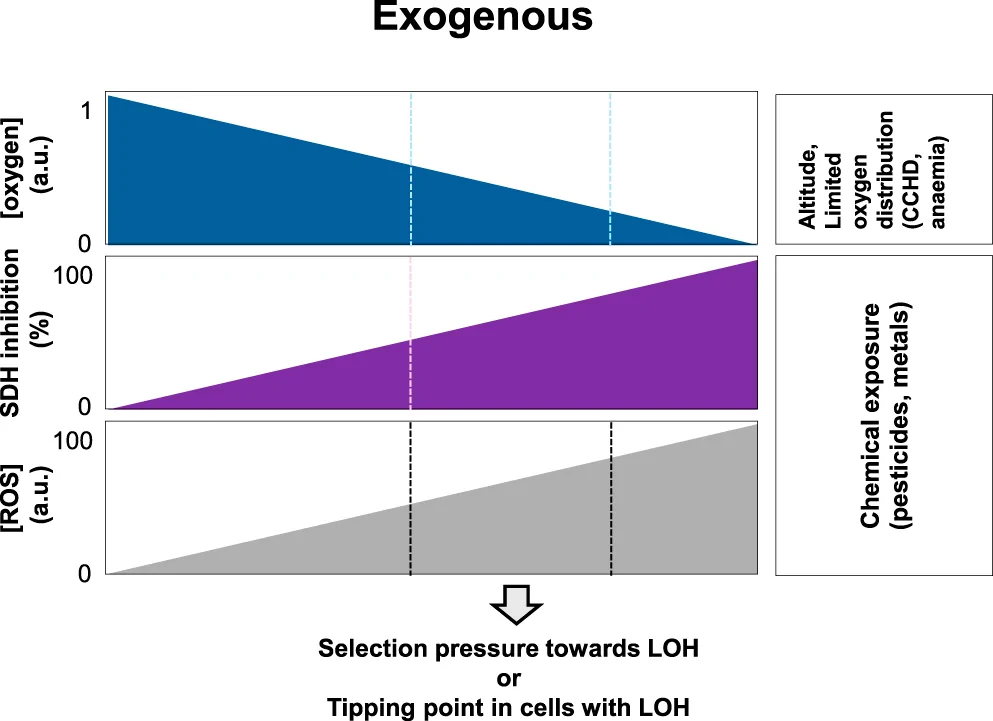
Model depicting exogenous factors influencing tumourigenesis in *SDHx* PV carriers. Hypoxia, SDH inhibition in conjunction with increasing succinate levels, and reactive oxygen species (ROS) may increase the selection pressure towards loss of the remaining *SDHx* wild-type allele as a transformative event, or create conditions in which cells with LOH experience a tipping point due to metabolic/redox pressure. The vertical lines indicate potential optimal ranges for tumourigenesis. CCHD – congenital cyanotic heart disease. LOH – loss of heterozygosity.

The incidence of PPGLs has increased over the past decades (1), reflecting more frequent incidental imaging and genotype-driven surveillance imaging, but potentially also an increase in population exposure to environmental factors (Fig. 2). Such factors include succinate dehydrogenase inhibitors (SDHi), which are fungicides widely used for the preservation of cereal seeds, fruits and vegetables, viticulture, arboriculture, ornamentals, public lawns, golf courses, and so forth. Population exposure may occur in proximity to areas where SDHi are used: in a study measuring approximately 100 pesticide residues across 47 sites, pesticides were found in 98% of sites, and two SDHi, fluopyram and fluxapyroxad, were detected in 69% and 68% of the sites, respectively (44). Inter-species conservation between amino acid sequences of SDH subunits is very high, as demonstrated among 22 organisms, including humans (45). Therefore, the potential impact of SDHi exposure on humans, and especially those who are genetically predisposed to developing SDH-related PPGLs (*SDHx* PV carriers), requires further investigation. *In vitro* studies carried out with various experimental models have shown that SDHi exposure increases the level of succinate in cellular media and induces a classical Warburg-like metabolic reprogramming that is also well documented in SDH-related tumours and associated with transcriptomic and morphological changes promoting cell migration and invasion (46).

Furthermore, the herbicide glyphosate, also predicted to inhibit SDH (47), was shown to increase oxidative stress and neuronal cytotoxicity in a human neuroblastoma-derived cell line, a model used as a surrogate for PPGLs due to the shared neural crest origin with neuroblastoma (48). Children exposed to pesticides showed elevated markers of oxidative stress, DNA damage, and pro-inflammatory features (49). Besides pesticides, exposure to metals, both essential and non-essential, contributes to increased oxidative stress, which is aggravated in the presence of catecholamines (50). Most tissues in which PPGLs arise produce catecholamines and may be exposed to higher ROS levels during chronic metal exposure. The resulting increase in DNA damage may cause not only loss of heterozygosity in individuals with a genetic predisposition, but also additional genetic events required for full oncogenic transformation. A recent study regarding neurofibromatosis type 1 supports such a hypothesis, in which the presence of a second *NF1* hit was shown to be necessary but not sufficient to induce cell transformation *in vivo* (51). Whether this also applies to *SDHx* PV carriers is unknown, but in this case, the additional metabolic or redox pressures exerted on cells carrying homozygous mutations might create a tipping point.

To assess the risks associated with SDHi exposure in humans, a recent feasibility study established the protocol for a matched case–control epidemiological study investigating the relationship between SDHi fungicides and the risk of PPGLs in subjects carrying *SDHx* PVs. Following the publication of a proof-of-concept study conducted at a single centre that included 50 patients with PPGLs due to *SDHx* PVs (cases) and 50 asymptomatic *SDHx* PV carriers (matched controls), this study is now being expanded (29). Results are expected to provide a clearer picture of the involvement of pesticides in the initiation of tumour development in *SDHx* PV carriers.

Another factor with considerable evidence for promotion of PPGL risk is sustained exposure to low oxygen or hypoxia (Fig. 2). Observations of enlarged carotid bodies – the oxygen-sensing chemoreceptor ‘organ’ – at an elevated altitude first implicated environmental influences in PPGL formation (52). It is now well documented that the carotid body is a common site of paraganglioma development in patients with and without an underlying genetic predisposition (28). Association of PPGLs with medical conditions that cause hypoxia in the body, such as cyanotic congenital heart or sickle cell disease and other haemoglobinopathies, further alludes to hypoxia as a risk factor for PPGL development (53, 54). Genetic investigations identified that accumulation of protein-stabilising mutations in the hypoxia-inducible factor gene *EPAS1* will eventually lead to PPGLs through a process of genetic adaptation to low oxygen (55). It is unknown, however, which level of hypoxia or duration of exposure is pathogenic. To complicate matters, the risk may vary depending on the variant of *SDHx* and other physiological differences and morbidities restricting efficient oxygen distribution in the body. Hence, it is difficult to advise patients on lifestyle choices, such as frequency of aeroplane travel, high-intensity endurance exercise, diving, or prolonged stays at high altitude locations.

Perhaps surprisingly, there is little evidence that cigarette smoking increases the risk of PPGLs, either with or without a hereditary predisposition. Although smoking may increase catecholamine secretion from PPGLs acutely (56), it does not appear to increase the risk of hypertension in patients with PPGLs (57), nor mortality or in-hospital outcomes from phaeochromocytoma-associated hypertensive emergencies (58). Unlike the well-described effect of altitude on carotid body hyperplasia noted above, the effect of smoking on the carotid body has been inconsistent, with some studies showing an increase in carotid body volume (59, 60) and others showing no change in size or enhancement (61, 62). To our knowledge, there are no published data on smoking-associated risk of PPGLs in *SDHx* PV carriers. Moreover, a smoking mutational signature was notably absent in genomic analyses of *SDHB*-related PPGLs (11). Despite the lack of strong evidence, but in accordance with general health principles, expert guidelines caution against cigarette smoking in subjects with *SDHx* PVs (63).

Tissue specificity for tumourigenesis from *SDH* PVs is not understood: why does *SDH* haploinsufficiency give rise to tumours in chromaffin cells but also sometimes renal cells and gastrointestinal stromal cells? Recently, microbial metabolites, such as from the gut microbiome, have been discussed as epigenetic and immune modulators that may impact tumourigenesis (64). Succinate is produced in substantial amounts by gut bacteria and can impel inflammatory bowel diseases: a shift in the balance of succinate-producing vs succinate-metabolising bacteria has been described, with inflammation mediated by subsequent increased interleukin-9 secretion from T helper cells (65). It is not inconceivable that succinate-driven inflammation may play a role in the development of GISTs in carriers of *SDHx* PVs. On the other hand, succinate produced by early-stage tumour cells may influence the microbiome composition with downstream effects on tumour cell proliferation. Microbiomes that alter metabolic features of the microenvironment were shown to influence colorectal cancer proliferation and immune evasion, and dietary interventions were suggested as a promising strategy (66). Moreover, microbiota differed between GISTs and benign microGISTs, with an enrichment of sugar metabolism demonstrated in the microbiome of GISTs (67). Besides usual advice to eat healthily, there is no evidence for specific dietary adjustments in asymptomatic people that would ‘optimise’ the gut microbiota, as this is not yet defined sufficiently for clinical implementation. Overall, there is not yet sufficient evidence about environmental risk factors to introduce recommendations to clinical practice.

## Conclusion

The risk of tumour development in carriers of *SDHx* PVs is governed by intrinsic factors, including the affected SDH subunit and the nature of the variant that also determines tissue-specific susceptibility to PPGL development. In addition, *SDHx* haploinsufficiency leads to adaptive changes in some cell types within the body. One consequence of these adaptations during embryogenesis is a higher transmission rate of the allele carrying the *SDHx* PV. On the other hand, blood cells from *SDHx* carriers show altered metabolic characteristics consistent with adaptation to either elevated succinate or the Warburg effect (i.e. anaerobic glycolysis). At this point, however, it is unclear if (and how) these influence the risk of tumour development. Exogenous factors, including low oxygen, and environmental pollutants, such as pesticides, are suspected to exert selective pressure towards loss or silencing of the wild-type allele in carriers of *SDHx* PVs. Especially for hypoxia, the association with PPGL development is well documented. Today, it is still difficult to translate current knowledge into recommendations for carriers. Future research may focus more on measures that have the potential to lower the risk of tumour development in carriers of *SDHx* PVs.

## Declaration of interest

The authors declare that there is no conflict of interest that could be perceived as prejudicing the impartiality of the work reported.

## Funding

SR received funding from the Paradifference Foundation. RCB received funding from Perpetual (Hillcrest Foundation).

## AI disclosure

No generative artificial intelligence (AI) tools were used in the preparation of the written or visual content of this manuscript.
